# Prosthetic treatment of women with lower limb absence during pregnancy & the postpartum period: A chart review

**DOI:** 10.33137/cpoj.v8i1.45142

**Published:** 2025-07-16

**Authors:** B.M Pousett, D Cumming, C Phillips, F Azhari, C MacKay

**Affiliations:** 1 Barber Prosthetics Clinic, Vancouver, Canada.; 2 Rehabilitation Sciences, Faculty of Medicine, University of British Columbia, Vancouver, Canada.; 3 West Park Healthcare Centre, University Health Network, Toronto, Canada.; 4 Department of Mechanical and Industrial Engineering, University of Toronto, Toronto, Canada.; 5 Department of Physical Therapy, Temerty Faculty of Medicine, University of Toronto, Toronto, Canada.; 6 School of Rehabilitation Therapy, Queen's Health Sciences, Queen's University, Kingston, Canada.

**Keywords:** Lower Limb Absence, Prosthetics, Perinatal Period, Survey, Pregnancy, Prosthetic Treatment, Rehabilitation, Postpartum Period, Women, Amputation, Prosthesis, Prosthetist

## Abstract

**BACKGROUND:**

Little information is available for women with lower limb absence (LLA) and their prosthetists regarding expectations for prosthetic treatment during and after pregnancy. A main concern is prosthesis use and what adjustments may be required to sustain mobility.

**OBJECTIVES:**

This study examines the prosthetic treatment of women with LLA to understand what specific prosthetic interventions occurred during the perinatal period and to gather information from the prosthetists regarding key learnings to be shared with others.

**METHODOLOGY:**

This study was a retrospective review of clinical records for women with LLA who received prosthetic care across Canada. Between January – May 2023, all 19 women who participated in a previous study on LLA and pregnancy consented to have their prosthetist contacted. Prosthetists were asked to complete a structured survey documenting appointment details, socket and alignment adjustments made during the perinatal period and key learnings in providing care to this population.

**FINDINGS:**

15 prosthetists were contacted to complete surveys for the 19 participants. Reviews of clinical records were completed between April – August 2023 by 7 prosthetists covering 18 pregnancies from 11 women with LLA (two bilateral transtibial, two unilateral transtibial, four unilateral transfemoral, and three unilateral rotationplasty). Socket adjustments were required in 11/18 pregnancies with common methods including circumferential stretching and localized adjustments. Alignment adjustments to existing sockets were only required in two pregnancies. Additional sockets were required in six pregnancies when the existing socket could no longer be adjusted to achieve comfort, most often during the first six months of pregnancy (the first or second trimester). Everyone who had a socket adjustment during pregnancy required additional socket adjustments or new sockets in the postpartum period. Prosthetists observed wide variations in physiological changes and prosthetic fit during the perinatal period and shared prosthetic management techniques to address residual limb volume changes. A data collection framework was also proposed to support the ongoing collection of this data to include a wider diversity of women and experiences.

**CONCLUSION:**

A wide range of prosthetic treatment interventions may occur during pregnancy and the postpartum period. While prosthetists and women with LLA can anticipate that socket and alignment changes may be necessary, sometimes none are required. By preparing for potential fluctuations in prosthetic fit and addressing each individual's needs, prosthetists can help minimize disruptions to mobility throughout pregnancy.

## INTRODUCTION

Pregnant women with physical disabilities often face a variety of challenges to receiving high quality perinatal care due to a lack of awareness and education among medical professionals.^1,2^ Limited information is available for both women and health care providers (HCPs) about the interaction between disability and pregnancy and about the needs of women living with disability throughout the perinatal period.^1–5^ This may be detrimental to care, as women have reported benefiting substantially from care where HCPs anticipated and carefully managed potential complications or challenges throughout their pregnancy.^3^ However, to facilitate this level of care, effort is needed to address the gap in HCPs’ education about disability and pregnancy, so that they can offer evidence based care.^1,5^

A notable example of this information gap pertains to pregnant women with lower limb absence (LLA), as there is little existing research on their experiences.^6,7^ Pregnant women experience changes to their body composition and center of mass throughout their pregnancy which can have impacts on their balance and mobility.^8–10^ These changes are typically largest in the second and third trimesters and begin to reverse postpartum, but can fluctuate.^9,10^ Pregnant women with LLA experience many of the same physical changes throughout the pregnancy and postpartum period, and reported fluctuations in limb volume and, balance, in addition to increases in pain and fatigue.^11,12^ However, these changes have particular significance for pregnant women with LLA as previous studies found 84% of individuals with LLA use prostheses as their primary form of mobility, for an average of 12.5 hours a day.^13^ As prostheses are fit very intimately to the wearer's body and are sensitive to volume changes, these changes can negatively impact prosthetic fit, mobility, physical function, balance, and the ability to perform daily tasks.^12,14^ Challenges with prosthesis use can require pregnant women to use other forms of mobility during the perinatal period, such as crutches or wheelchairs.^3,11,14^ This leaves women with LLA with many questions surrounding what to expect in regards to their mobility and prosthesis use when considering having children.^12,15^

Current evidence on prosthetic management during the perinatal period is scarce. A systematic review of pregnancy experience for women with LLA included several case series; however, very few commented on prosthesis use or mobility.^16^ A recent clinical consensus guideline acknowledges that, during pregnancy, female patients with transfemoral amputations may need socket adjustments to accommodate volume gain, trimline modifications to allow for comfortable sitting, and alignment adjustments to account for center of mass changes.^15^ The guideline also states that new sockets may be necessary but provides no concrete evidence. In one case study, a woman with a transfemoral amputation visited her prosthetist ten times due to physiological changes during a pregnancy,^12^ while for most people with LLA, it is common to visit a prosthetist once every six months unless problems arise.^17^ This case study found that, during pregnancy, socket comfort, gait speed and functional mobility declined, and that socket adjustments could increase comfort and decrease pain. However, the specific details of the nature of these adjustments were not provided.^12^ A recent study in Canada of mobility outcomes for people with LLA during pregnancy reported that among highly active daily prosthesis users, 64% experienced changes in limb size and prosthesis comfort. Additionally, 71% of those who used their prosthesis during pregnancy decreased their prosthesis use due to limb swelling and over a quarter reported using a wheelchair or gait aid during pregnancy for mobility.^14^ While this study^14^ documents the challenges with limb volume change and prosthesis use, there is no information provided on the specifics of the prosthetic management.

The limited evidence has left both health care providers and women with LLA without adequate information on prosthetic management during the perinatal period.^6,7^ Therefore, this study seeks to explore the prosthetic treatment of women with LLA throughout the perinatal period (defined as the period from the beginning of pregnancy to one year after delivery). We sought to understand which specific prosthetic interventions were used by prosthetists (including socket and/or alignment adjustments, new sockets, etc.) during the perinatal period. We have used the responses to these questions to propose a data collection framework for prosthetists providing care to pregnant women with LLA. This framework identifies key areas where evidence is lacking and suggests methods for collecting the necessary data.

## METHODOLOGY

We conducted a retrospective review of clinical records from prosthetic clinics across Canada regarding prosthetic treatment for women with LLA during the perinatal period. The review was conducted from April to August 2023. Ethics approval was provided by the Research Ethics Board at the University of Toronto.

### Participants

**Figure 1:**
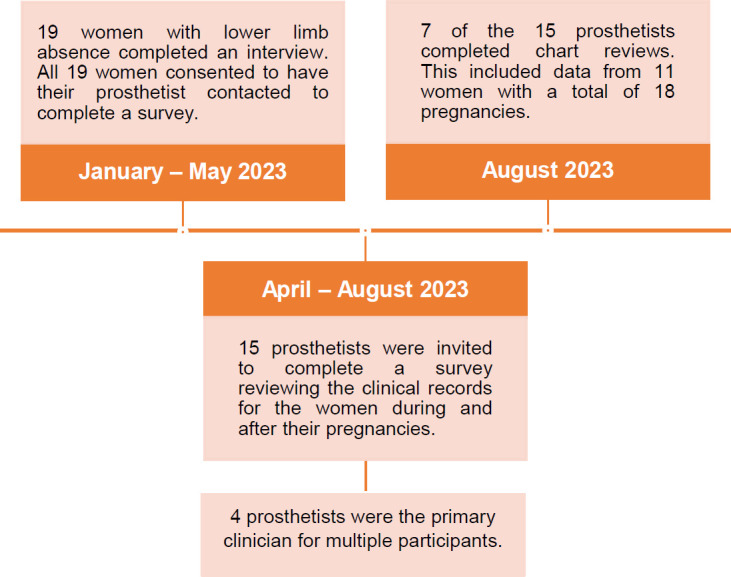
Participant recruitment and data collection process.

A previous study examined the physical and psychosocial experiences of women with LLA during the perinatal period.^7,11^ To be eligible for the previous study, women had to live in Canada, have a LLA, and have been pregnant in the last 10 years. All women who participated in that study were asked if they consented to have their prosthetist contacted to provide a chart review for the dates they were pregnant in the previous 10 years. All 19 women who previously participated consented and provided the name of their prosthetist and the start and end dates (month and year) of each pregnancy. The 15 corresponding Canadian prosthetists were invited to participate via email (four of the prosthetists had seen two participants). Of the 15 invited prosthetists, 7 completed chart reviews. Prosthetists represented clinics in 3 provinces (Alberta = 4, British Columbia = 2, Ontario = 1). They were given the names of the patients who consented and the date range of each pregnancy and asked to extract data from clinical chart to answer questions in a survey format for each pregnancy. See **Figure 1** for a detailed visual of the recruitment and data collection process.

### Survey

The structured survey (**Appendix A**) was developed specifically for this study by authors BMP and FA, one is a prosthetist, and one is a woman with LLA who has been pregnant. It was piloted with a prosthetist who had experience providing care to women with LLA during pregnancy. The survey included a detailed prosthetic prescription for each woman as well as how many appointments they had during their pregnancy and what was done at each appointment (including socket adjustments, alignment adjustments, socket replacements, new componentry required, etc.). The survey also asked if any adjustments occurred in the postpartum period (defined as being one year following pregnancy). Prosthetists were given the opportunity to share what they learnt about working with women during the perinatal period and what information they wished was in resource material.

### Data Analysis

Quantitative data (such as number of appointments, type of prosthesis, level of amputation, and nature of the adjustment) was analyzed using descriptive statistics. Open text boxes were analyzed using conventional content analysis, where we reviewed the data, inductively developed codes from key concepts, grouped the codes together into categories, and summarized the findings for each category.^18^ These text boxes were also used to provide context to the data captured by quantitative methods.

## results

Chart reviews of 18 pregnancies from 11 women with LLA were included. This corresponds to 58% of women who consented to have their prosthetists contacted. Six women had one pregnancy included, three women had two pregnancies included, and two women had three pregnancies included. Levels of amputation included bilateral transtibial amputation/ankle disarticulation (n = 2), unilateral transtibial amputation (n = 2), transfemoral amputation (n = 4), and rotationplasty (n = 3). The average number of appointments during pregnancy was 4.2 (SD: 3.6), with a range of 0 to 12. Suspension and interface types can be found in **Table 1**.

**Table 1:** Summary of prosthetic treatment for each woman included in the chart review.

ID	LLA Level	Interface/Suspension System	Pregnancies (n)	Appts (n)^*^	During Pregnancy			During Postpartum	Notes
					Socket Adjustments Adjustments	Alignment Adjustments (Trimester)	New Socket (Trimester)	Adjustment Made After Pregnancy	
A	BTT	Socks/Anatomical/Supra - condylar	1 of 1	3	None	None	No	None	Fit diagnostic socket with 4 ply socks in T1 (before pregnancy known). Socks were supplied.
B	RP	Socks/Anatomical/Supra-condylar	1 of 2 (only 2^nd^ included)	2	None	None	No	None	Provided shrinker socks to use if needed. Patient asked prosthetist for peer support connections after finding no resources.
C	RP	Socks/Cuff/strap	1 of 1	1	1 localized adjustment: removed padding on dorsum of foot (T3)	None	No	New socket required as limb was smaller than before pregnancy	
D	TF	Silicone Liner/Locking Liner - Pin	2 of Unknown	5	3 circumferential adjustments: stretched socket globally over cast. (once in T2 on first	None	Yes (T2)	Alignment – did not specify how	
				8	2 circumferential adjustments: stretched socket from first pregnancy globally over cast (T1, T2)	Lengthened prosthesis (T3)	Yes 2 sockets (both T2)	Shortened prosthesis. Refit previous socket	Switched to socket from first pregnancy early in T1 before any adjustments done
E	BTT	Gel Liner/Suction Sleeve	1 of 1	10	4 localized adjustments: adjusted fibular head via heating (T1), adjusted medial hamstring and anterior distal tibia by heating and grinding (T1), adjuster posterior trimline via grinding (T2), adjusted proximal anterior tibia via grinding & medial hamstring via heating (T2)	None	Yes (T1)	Pressure relief – did not specify where or how.	
F	TF	Silicone Liner/Seal-In Liner	1 of 1	3	1 circumferential adjustment: stretched proximal 2/3 of socket (T1)	None	Yes (T1). Refit a previous check socket.	Unknown. Did not follow up with that clinic.	
G	TT	Gel Liner/Locking Liner – Pin	2 of 3 (2^nd^ & 3^rd^ included)	9	2 localized adjustments: adjusted knee condyles via grinding (T1, T1). 1 circumferential adjustment–stretched 2^nd^ new socket distally (T3).	None	Yes (2 sockets) (T1 & T2)	New socket	New sockets made after localized adjustments.
				3	None	None	No	None	
H	TF	Skin/Skin fit Suction	3 of 3	12	5 localized adjustments: adjusted medial trimline by grinding (T1), adjusted medial trimline by grinding and heating (T1), added padding to lateral socket (T2), added padding to anterior socket (T2), removed padding from lateral socket (T3)	None	No	Added padding to all 4 walls of socket.	New socket was fit before pregnancy was known, adjustments may have been part of routine fitting.
				6	1 localized adjustment: removed padding from medial, lateral and anterior socket (T2)	None	No	Replaced padding that was removed during pregnancy	Prosthetist would have liked to increase socket volume in last month of pregnancy but didn't want to make changes that could not be easily reversed.
				7	1 localized adjustment: adjusted distal lateral femur by grinding (T1). 1 circumferential adjustment–ground proximal socket as much as possible before risking socket failure. (T2)	None	No	Added additional padding as volume decreased.	
J	TF	Socks/Anatomical/Supracondylar & Cuff/Strap	3 of 3	2	None	None	No	None	Lengthened waist belt (T2)
				1	None	None	No	None	
				1	None	None	No	None	
K	RP	Neoprene Sock/Cuff/Strap	2 of 2	1	1 circumferential adjustment: lengthened thigh corset (T2)	None	No	Shortened thigh corset	
				0	None	None	No	None	
L	TT	Gel Liner/Locking Liner - Pin	1 of 1	2	Needed (not made due to funding)	Shift socket medially to widen base of support (T3)	Yes (T3) (not made due to funding)	None	Patient opted to persevere with poorly fitting socket

* Number of Appointment.

**Level of Amputation: BTT** = Bilateral Transtibial or Ankle Disarticulation, **RP** = Rotationplasty, **TF** = Transfemoral, **TT** = Transtibial.

**Trimester: T1** = first trimester, **T2** = second trimester, **T3** = third trimester.

**Localized:** Adjustment done in a specific area.

**Circumferential:** adjustment done over entire circumference of the socket.

### Socket Adjustments

No socket adjustments were needed in 7/18 pregnancies. Two women (A*: bilateral ankle disarticulation with one pregnancy, J: transfemoral amputation with three pregnancies) required no socket adjustments in any pregnancy. Two women (K: rotationplasty, G: transtibial amputation) had adjustments made in their first pregnancy but no adjustments made in their second pregnancy. One woman (B: rotationplasty) had no adjustments made in her second pregnancy but the chart review for the first pregnancy was not completed by the prosthetist.

Socket adjustments were needed or made in the remaining 11/18 pregnancies to adjust sockets as described in **Table 1**. Circumferential adjustments were common (n = 5: D, F, G, H & K; 3 transfemoral, 1 transtibial, 1 rotationplasty), especially for those with transfemoral sockets (n = 3: D, F, H), and were done via heating and stretching the socket, removing existing padding or grinding. A transtibial socket (G) was also circumferentially adjusted via heating and stretching and one rotationplasty prosthesis (K) had the corset made larger. Localized adjustments were also common (n = 4: C, E, G & H rotationplasty, bilateral transtibial, transtibial, transfemoral), typically via grinding to remove material. Areas frequently adjusted were fibular head (transtibial), medial hamstring (transtibial), anterior distal tibial (transtibial), anterior proximal tibia (transtibial), knee condyles (transtibial), dorsum of foot (rotationplasty) and medial wall (transfemoral). For one woman using a transfemoral waist belt for suspension, the strap was made longer.

In regard to when adjustments were done, localized adjustments tended to be early in pregnancy, during the first or second trimester, while circumferential adjustments were spread out across all trimesters.

### Alignment Adjustments

Alignment adjustments on existing sockets were only done in 2/18 pregnancies – once to lengthen the prosthesis (D: transfemoral – 3rd trimester) and once to shift the socket medially to widen the base of support (L: transtibial – 3rd trimester). The prosthesis that was lengthened was shortened again in the postpartum period. One clinician measured the changing center of mass of their patient with a transfemoral amputation (J) using the LASAR Posture (Ottobock) and found it moved 29 mm forward from baseline to 37 weeks pregnant.

### At Home Management

At home management was rarely documented. Socks and shrinkers were each provided to one patient (A, B). One woman (A) was fit with diagnostic transtibial/ankle disarticulation sockets that were too large right after she found she was pregnant, in anticipation of limb volume gain. The prosthetist reported that this worked well and was able to support socket fit management by providing thinner socks as the pregnancy progressed.

### New Sockets

Twelve pregnancies did not require a new socket, and six pregnancies did require a new socket (D x 2, E, F, G, L) (**Table 1**). New sockets were required when existing sockets could no longer be adjusted to comfortably fit. In one situation, a new socket was required but not made due to the inability to receive funding approval. When new sockets were fit, dynamic alignment was done as is standard clinical practice. New sockets were most often made in the first or second trimester. One prosthetist commented that a new socket was needed but the patient opted to persevere with their existing socket (L).

### Postpartum Period

After pregnancy, nothing was done in the 7/18 pregnancies who had no adjustments made during pregnancy. Two women (C, G) required a new socket post-delivery as their current socket no longer fit appropriately: one who had adjustments made during pregnancy (C: rotationplasty), and the other who had both adjustments and 2 new sockets made during pregnancy (G: transtibial). One woman (D: transfemoral) required adjustment to her alignment after her first and second pregnancies and as after pregnancy she switched from a larger socket that was fit during pregnancy, to a previously worn socket that was smaller. Three women (E, H, K) required socket adjustments to reverse adjustments made during pregnancy. All women who had adjustments during pregnancy also had adjustments after pregnancy.

### Learnings & Take Aways

Prosthetists reported key learnings from providing prosthetic care to women with LLA during the perinatal period. In the prenatal period, prosthetists reported observing a wide range of physiological changes and changes to prosthetic fit. Some found that their patients managed without any changes to their prostheses but that it depended on the individual and level of amputation.

Others reported the difficulty of managing prosthesis use during pregnancy due to changes with weight, limb volume, socket pressure, and the location of the center of mass. A wide range of specific learnings were reported with prosthetists reporting different observations about the patient they worked with. These included that “*volume changes are greatest in the 3rd trimester*”, “*adjustments are needed more often for individuals with transfemoral amputations than transtibial amputations*”, “*patients with congenital limb loss have less volume concerns and need for adjustments*” and “*weight gain impacts the thigh not the foot for people with rotationplasty amputations*”. However, these observations are not necessarily supported by the aggregated data. For example, we did not see the most adjustments or new sockets in the third trimester to match the observation that this is when there were the greatest volume changes. Also, for people with rotationplasty amputations, we saw examples of both the foot and the thigh section needed to be adjusted. One prosthetist reported that “*setting up a schedule to meet regularly [over the pregnancy] was a good way to keep small problems small*”.

In the postpartum period, prosthetists reported a wide range of learnings; some found that volume changes reversed quickly, while some found it took approximately three months to get back to a pre-pregnancy socket fit.

### Managing Volume Change

Prosthetists reported their key learnings surrounding finding success with several different volume management techniques, including:

- Using interfaces that can be easily modified to allow maximum adjustment (e.g., flexible inner liners, Pe-Lite liners)
- Heating and stretching sockets
- Holding on to previous sockets so that one has multiple sockets of different sizes to help accommodate volume changes during and after pregnancy

Five prosthetists commented that making “future-proof” sockets that allow for volume adjustment is helpful. Ways of doing this include making sockets larger, making sockets with liners that can be removed, making sockets with padding that can be removed, etc.

### Helpful Resources

One prosthetist reiterated that there are no resources available for pregnant women with LLA but that peer support and advice from other women with LLA who have been pregnant can be helpful. The remaining prosthetists did not comment on any helpful resources.

## DISCUSSION

This study presents the experiences of prosthetists providing care to women with LLA during and after pregnancy, and the wide range of prosthetic treatment interventions that occur. The review of clinical records includes women with a wide range of levels of amputations, including both ankle disarticulation and rotationplasty amputations, which are infrequently included in research. Some women did not require any prosthetic appointments or adjustments during pregnancy, while others visited their prosthetist frequently. These findings spanned all levels of amputation, and no level-specific recommendations can be made due to the small number of participants and the wide range of experiences. However, for those who did not require any adjustments over their pregnancy, many, though not all, of these were women in their second or third pregnancies and had sockets that had previously been adjusted. Prosthetists should be diligent—particularly in first pregnancies—to anticipate and address any prosthesis issues that arise, while also being aware that women may require no adjustments in pregnancy. As always, it is important to treat each patient and their unique situation individually.

Prosthetists demonstrated a large toolkit of possible prosthetic treatment interventions when working with women whose pregnancy-related changes did require such interventions. These most often included global socket adjustments, localized socket adjustments, and new sockets. While limb volume change is reported to peak later in pregnancy,^9,10^ adjustments did not follow the same trend. Localized adjustments tended to be made earlier in pregnancy (in the first and second trimesters) and circumferential adjustments were made approximately evenly throughout all trimesters. All women who required a socket adjustment during pregnancy had adjustments after pregnancy as well, demonstrating that it is important to reassess prosthetic fit and function in the year after pregnancy.

While not frequently documented within the clinical charts, one prosthetist reported in the open-ended questions that they provided supplies for at-home management. In the previous phase of this research, women with LLA frequently discussed how their prosthetists provided them with education and supplies for at-home management that helped them throughout their pregnancies.^11^ This discrepancy highlights the importance of the at-home management strategies for women with LLA and emphasizes that prosthetists need to ensure they provide education on appropriate at-home management strategies in the future.

We know that the center of mass changes throughout pregnancy, which may impact prosthesis function.^12,15^ Alignment is sensitive to the location of the center of mass; however, no one in our study reported making an alignment adjustment in the anterior-posterior plane, and only one prosthetist reported making an adjustment in the mediallateral plane. It is unclear if this is because the center of mass moves so slowly that the body makes other adaptations and therefore prosthesis function is not impacted in a way requiring alignment or componentry adjustments. This is something that needs to be more closely noted in the future.

New sockets were required for those with residual limb volume and shape changes beyond what could be accommodated for in an existing socket. In this study, most of the sockets required were made in the first or second trimester, even though the weight gain and limb volume change tend to be the largest later in pregnancy, in the second and third trimester. While our data cannot explain this discrepancy, it appears the prosthetists included in this chart review were proactive about keeping sockets fitting comfortably early on in pregnancy and mentioned several strategies that allowed them to accommodate for volume increases over pregnancy. Our previous research has found barriers to accessing prosthetic care, including scheduling constraints, funding availability and the time it takes for a socket to be made may limit women from accessing prosthetic care.^11^ Women who need adjustments later in pregnancy but choose not to seek prosthetic care were not captured in this chart review study, except for the one woman whose prosthetist noted that a socket was required but not supplied (L) and one prosthetist who commented that they would have liked to further increase the socket volume in the last month of pregnancy but didn't want to make a non-reversable change (H). Future research should look specifically into when new sockets are required in pregnancy, regardless of if treatment is sought.

Overall, prosthetists demonstrated strategies similar to those mentioned by women with LLA, notably that planning ahead is key to successfully managing prosthesis fit throughout pregnancy.^7^ These planning-ahead techniques included having conversations early, making adjustable interfaces, using thermoplastic check sockets that can more easily be stretched or adjusted, keeping old sockets for women of childbearing age in case they are needed, making sockets too big early in pregnancy, and using techniques to future-proof sockets.

### Data Collection Framework

The information gathered in this study sets the foundation for a larger data collection framework (**Appendix B**). While the current study provides more information than previous studies on experiences of pregnant women with LLA regarding their prosthetic treatment,^12,14,16^ additional data is needed to provide a comprehensive overview, which would include the diversity of women and their specific prostheses. The framework should include details on socket adjustments, new sockets, alignment adjustments and at-home management. When documenting new sockets specifically, it should be noted when the new socket is identified as being needed, regardless of if it is supplied. Women and prosthetists may choose to tolerate a poorly fitting socket or switch to using other forms of mobility instead of investing the time and money required to make a new socket.

Overall, the format of the current survey seemed to have been satisfactory for the purposes of this study and the data was effective for describing their experience and informing future care. 47% of the 15 prosthetists who were invited to participate completed the survey. It is important to note that of 15 invited prosthetists, it was discovered that one prosthetist had passed away and two prosthetists had retired from their clinical practice. From the current review, data on socket adjustments and the provision of new sockets seemed straightforward to collect, while information on alignment and at-home management was less specific. In the future, attention should also be given to documenting alignment adjustments and to education on at-home management techniques. We may also need to explore different ways of asking these questions.

Data collection needs to continue to include information on patient demographics (e.g., cause of amputation, level of amputation, BMI, ethnicity, activity level, etc.) and pregnancy details (e.g., number of pregnancies, time in the pregnancy that adjustment is made, weight gain, etc.). In the current study, prosthetists hypothesized that the third trimester was the trimester most likely to require adjustments to accommodate for changes, and that women with congenital LLA have less limb volume and shape change issues; however, we do not have enough data to provide evidence for or against these speculations. In addition, it could be helpful to collect more details regarding the postpartum period, including details on how the socket fits in the 3–12 months after birth. In the current study, prosthetists commented that it takes approximately three months to get back to pre-pregnancy limb volume, despite women commenting it took up to 18 months,^11^ demonstrating the discrepancies between these two points of view. The proposed framework builds off of the existing chart-review survey and adds components which should be reported by both the patient and the prosthetist. Information from prosthetists should rely on objective adjustment data, while women with LLA can provide any additional contextual information. In the future, this data collection framework could be expanded to collect information on the experiential aspects of pregnancy from women with LLA as well.

While this study provides suggestions for the questions and format of a data collection framework, it does not identify a sustainable location. Perhaps this research could inform limb loss registries, which could expand to have a section dedicated to pregnancy and limb loss.^19^

### Limitations

While this study fills a gap regarding information for prosthetists caring for pregnant women, it was done in a Canadian context with a small subset of prosthetists. Given the variety of experiences of women with LLA during the perinatal period, a larger dataset is needed over a wider geographic area with more diversity of women and their prosthetic prescriptions. In addition, there may be other factors that influence prosthetic treatment such how well the socket was fitting prior to pregnancy or how often a particular patient typically requires adjustments to maintain a comfortable socket fit. We cannot make any claims regarding the correlation between level of amputation and prosthetic treatment due to the lack of other demographic factors (e.g. age, prosthetic componentry, activity level, prosthetic use, etc.). These demographic factors were not included in the scope of the current study but are included in the data collection framework so that future studies can make more robust conclusions and recommendations.

In addition, this study was a survey of clinical records with a few additional questions added to gather the ideas of the prosthetists. The time between seeing the patient and completing the review could have been up to 10 years, which may impact recall if prosthetists tried to supplement data written in the chart. In the future, it would be ideal to complete the review in the 12-18 months after pregnancy.

## CONCLUSION

This study provides valuable information regarding the prosthetic treatment of women with LLA during the perinatal period. It highlights the diverse experiences of prosthetists providing care to these women and the wide range of prosthetic interventions that were found appropriate. It also provides suggestions for future data collection to gather additional information to address this critical gap in knowledge. Given the findings from this study, we can begin to better educate women with LLA and their prosthetists regarding what to expect during and after pregnancy and the range of experiences they may encounter. This can also provide information to prosthetists to ensure they have the tools and knowledge to provide their patients with as smooth a journey as possible. By providing information on the range of experiences and prosthetic management techniques, we can give women with LLA and their prosthetists the information they need plan ahead for a variety of scenarios, leading to decreased anxiety about uncertainty regarding how to manage limb volume change and prosthesis fit during the perinatal period.

## Footnotes

* Letters refer to subject identifiers listed in Table 1.

## Appendix B

### Section 1A: Patient Details: General

***To be completed by the prosthetist or patient, depending on if this information is documented in the clinical records.***

Birth month and year:

Race or racial background: (check all that apply)

- Black (African, African Canadian, Afro-Caribbean descent)
- East Asian (Chinese, Japanese, Korean, Taiwanese descent)
- Indigenous (First Nations, Inuk/Inuit, Métis descent)
- Latin American (Hispanic or Latin American descent)
- Middle Eastern (e.g., Afghan, Egyptian, Iranian, Kurdish, Lebanese, Turkish)
- South Asian (e.g., Bangladeshi, Indian, Indo-Caribbean, Pakistani, Sri Lankan)
- Southeast Asian (Cambodian, Filipino, Indonesian, Thai, Vietnamese, or other Southeast Asian descent)
- White (European descent)
- Other
- Do not know
- Prefer not to answer

Side of Amputation: (check all that apply)

- Right
- Left

**For each side with an amputation:**

Level of Amputation:

- Symes/Ankle Disarticulation
- Transtibial
- Knee Disarticulation
- Transfemoral
- Hip Disarticulation
- Rotationplasty
- Hemipelvectomy
- Other:

Cause of amputation:

- Vascular/diabetes
- Trauma
- Infection
- Cancer
- Congenital
- Other: ________

### Section 1B: Patient Details at time of Pregnancy

***To be completed by the prosthetist:***

**For each side with an amputation, what was the prosthetic prescription at the time of pregnancy?**

Prosthetic interface:

- None (skin)
- Socks
- Pelite/foam Liner
- Gel Liner
- Silicone Liner
- Urethane Liner
- Neoprene Sock
- Other: ________

Suspension mechanism:

- Skin Fit Suction
- Anatomical/Supracondylar
- Cuff/Strap
- Tension Sleeve
- Suction Sleeve
- Locking Liner – Pin
- Locking Liner - Lanyard
- Seal-In Liner
- Elevated Vacuum
- Other: ___________

Socket Design:

- Window (obturator)
- Total surface bearing (Transtibial)
- Patella tendon bearing (Transtibial)
- Specific weight bearing (Transtibial)
- Hydrostatic (Transtibial)
- Thigh lacer
- Quadrilateral (Transfemoral)
- Narrow ML (Transfemoral)
- Ischial Containment (Transfemoral)
- Sub-ischial (Transfemoral)
- MAAS (Transfemoral)
- Other:____________

Foot:

Knee or side joints: (if applicable):

Hip joint (if applicable):

***To be completed by the patient:***

What was your activity level at the time of pregnancy?

- Low
- Moderate
- High
- Extremely high

Did you wear your prosthesis as your primary means of mobility for your entire pregnancy?

- Yes
- No

If no, when did you stop using it and what form of mobility did you use instead?

What was your pre-pregnancy weight?

### Section 2: Pregnancy Details

***To be completed by the patient:***

How many times have you been pregnant?

What was the anticipated due date? (This will be used to calculate in which trimester adjustments were done.)

What was the baby's birth date? (This question in combination with the question above can calculate the dates of pregnancy).

How much weight did you gain during each pregnancy?

### Survey Section 3: Appointments

***To be completed by the prosthetist:***

How many appointments did this patient have during their pregnancy? (Calculated as the 9 months before the due date to the birth date).

**For each appointment:**

What was the date of the appointment?

What was the goal of this appointment?

Were any socket adjustments made? (If yes, answer the questions below)

- What was the goal of the socket adjustment?
- Was pressure added or removed?
- What was the method of adjustment
  - Padding
  - Heating
  - Grinding
  - Other: ________
- Which location(s) was the adjustment made in?
  - Anterior distal tibia
  - Medial tibial flare
  - Posterior tibial area
  - Fibula
  - Knee condyles
  - Medial thigh
  - Lateral thigh
  - Anterior thigh (scarpas)
  - Posterior thigh
  - Other: _______
- Please describe the adjustment in as much detail as possible:

Were any alignment adjustments made? (If yes, answer the questions below)

- What was the goal of the alignment adjustment?
- Which of the following was done?
  - Socket flexion
  - Socket extension
  - Socket abduction
  - Socket adduction
  - Socket medial shift
  - Socket lateral shift
  - Socket posterior shift
  - Socket anterior shift
  - Knee internally rotated
  - Knee externally rotated
  - Foot inverted
  - Foot everted
  - Foot toed in
  - Foot toed out
  - Prosthesis lengthened
  - Prosthesis shortened
  - Other: _________
- Please describe the alignment adjustment in as much detail as possible:

Was a new socket indicated? (If yes, answer the questions below)

- Please describe why it was determined that a new socket was required.
- Will the new socket be made? If no, please describe why it will not be made (i.e., funding, time requirements, etc.)
- If the socket is not fitting properly, will the patient use alternative means of mobility? If so, what mobility devices will they use?

Were any consumables or components required at this appointment relevant to the pregnancy? (If yes, answer the questions below)

- Pease describe the consumables and components required at this appointment relevant to the pregnancy.

Was any education provided on at-home management strategies (e.g., wearing shrinker socks when not wearing the prosthesis, switching to thinner socks or liners, etc.)? If so, please describe.

### Section 3: Post-Natal Questions

***To be completed by the prosthetist:***

Please describe all the adjustments made in the year after pregnancy that revered adjustments made during the pregnancy, accommodated volume changes or involved the provision of a new socket.

### Section 4: General Questions

***To be completed by the prosthetist:***

Did you (the prosthetist) learn anything while treating this patient during pregnancy that you think may be helpful for other people treating pregnant individuals with lower extremity prostheses to know? If yes, please describe.

Is there anything else from treating this patient during this pregnancy that you would like to share with us?

## References

[1] Tarasoff LA. Experiences of women with physical disabilities during the perinatal period: A review of the literature and recommendations to improve care. Health Care Women Int. 2015;36(1):88–107. DOI: 10.1080/07399332.2013.815756 23998776

[2] Mitra M, Long-Bellil LM, Iezzoni LI, Smeltzer SC, Smith LD. Pregnancy among women with physical disabilities: Unmet needs and recommendations on navigating pregnancy. Disabil Health J. 2016;9(3):457–63. DOI: 10.1016/j.dhjo.2015.12.007 26847669 PMC4903955

[3] Long-Bellil L, Mitra M, Iezzoni LI, Smeltzer SC, Smith L. The impact of physical disability on pregnancy and childbirth. J Womens Health. 2017;26(8):878–885. DOI: 10.1089/jwh.2016.6157 PMC5576221 28661774

[4] Tarasoff LA. “We don't know. We've never had anybody like you before”: Barriers to perinatal care for women with physical disabilities. Disabil Health J. 2017;10(3):426–433. DOI: 10.1016/j.dhjo.2017.03.017 28404229

[5] Smeltzer SC, Mitra M, Iezzoni LI, Long-Bellil L, Smith LD. Perinatal experiences of women with physical disabilities and their recommendations for clinicians. JOGNN. 2016;45(6):781–789. DOI: 10.1016/j.jogn.2016.07.007 27619410 PMC5107149

[6] Hanna E, Donetto S. The pregnancy experiences of amputee women: a qualitative exploration of online posts. J Reprod Infant Psychol. 2021;43(3):1–11. DOI: 10.1080/02646838.2021.2004301 34789041

[7] Cumming D, MacKay C, Phillips C, Azhari F, Pousett BM. Resources, relationships, and resilience: The psychosocial experiences of women with lower limb absence during pregnancy and postpartum. Disabil Health J. 2024;17(3):1–6. DOI: 10.1016/j.dhjo.2024.101621 38582628

[8] Takahashi Y, Kaji T, Yasui T, Yoshida A, Yonetani N, Suzue N, et al. Ultrasonographic changes in quadriceps femoris thickness in women with normal pregnancy and women on bed rest for threatened preterm labor. Sci Rep. 2022;12(1). DOI: 10.1038/s41598-022-22467-8 PMC9582004 36261471

[9] Haddox AG, Hausselle J, Azoug A. Changes in segmental mass and inertia during pregnancy: A musculoskeletal model of the pregnant woman. Gait Posture. 2020;76:389–95. DOI: 10.1016/j.gaitpost.2019.12.024 31927359

[10] Widen EM, Gallagher D. Body composition changes in pregnancy: Measurement, predictors and outcomes. Eur J Clin Nutr. 2014;68(6):643–52. DOI: 10.1038/ejcn.2014.40 24667754 PMC4078736

[11] Pousett BM, Cumming D, Azhari F, Phillips C, MacKay C. The physical experiences of women with lower limb absence during pregnancy and postpartum: symptoms, prosthesis management & mobility. Disabil Rehabil. 2025;47(6):1587–1594. DOI: 10.1080/09638288.2024.2378234 39023179

[12] Kahle JT, Miro RM, Ho LT, Gagliardotto A, Swanson AE. Effect of pregnancy on anthropometrics, comfort, and functional performance for women living with transfemoral limb loss: Case report. Prosthet Orthot Int. 2023;48(3):315–319. DOI: 10.1097/pxr.0000000000000260 37498781

[13] Raichle KA, Hanley MA, Molton I, Kadel NJ, Campbell K, Phelps E, et al. Prosthesis use in persons with lower-and upper-limb amputation. J Rehabil Res Dev. 2008;45(7):961–72. DOI: 10.1682/jrrd.2007.09.0151 19165686 PMC2743731

[14] Bateman EA, Viana R, Sales D, Payne MWC. Pregnancy after amputation: A national survey of prosthetic and mobility outcomes in women with lived experience. Prosthet Orthot Int. 2024;49(2):179–184. DOI: 10.1097/pxr.0000000000000407 40228235 PMC11984547

[15] O'Brien E, Stevens PM, Miro R, Highsmith MJ. Transfemoral interface considerations: A clinical consensus practice guideline. Prosthet Orthot Int. 2023;47(1):54–59. DOI: 10.1097/pxr.0000000000000182 36450007

[16] Bateman EA, Frengopoulos C, Viana R, Payne MWC. Pregnancy after amputation: A systematic review of pregnancy experiences for women with lower extremity amputations. Am J Phys Med Rehabil. 2022;101(11):1066–75. DOI: 10.1097/phm.0000000000001949 35034056

[17] Hanger Clinic. Patient care manual: above-knee amputation [Internet]. Austin (TX): Hanger Clinic; [cited 2024 June 13]. Available from: https://hangerclinic.com/wp-content/uploads/patient-care-manual-above-knee.pdf

[18] Hsieh HF, Shannon SE. Three approaches to qualitative content analysis. Qual Health Res. 2005;15(9):1277–88. DOI: 10.1177/1049732305276687 16204405

[19] Limb Loss and Preservation Registry [internet]. McLean, VA: Limb Loss and Preservation Registry. [cited 2024 June 13]. Available from: https://www.llpr.org/

